# Supplementary material for: TMX4-driven LINC complex disassembly and asymmetric autophagy of the nuclear envelope upon acute ER stress
Source: Nat Commun. 2023 Jun 13;14:3497. doi: 10.1038/s41467-023-39172-3 (PMC10264389; doi:10.1038/s41467-023-39172-3)
Supplement: Supplementary file 1 — Supplementary Information [file 41467_2023_39172_MOESM1_ESM.pdf]

TMX4-driven LINC complex disassembly and asymmetric autophagy of the nuclear envelope upon acute ER stress

Marika K. Kucińska[1,2*], Juliette Fedry[3*], Carmela Galli[1], Diego Morone[1,4], Andrea Raimondi[1,5], Tatiana Soldà[1], Friedrich Förster[3], Maurizio Molinari[1,6]

[1]Università della Svizzera italiana (USI), Faculty of Biomedical Sciences, Institute for Research in Biomedicine, CH-6500 Bellinzona, Switzerland
[2]Department of Biology, Swiss Federal Institute of Technology, CH-8093 Zurich, Switzerland
[3] Structural Biochemistry, Bijvoet Center for Biomolecular Research, Utrecht University, 3584 CG Utrecht, The Netherlands
[4]Graduate School for Cellular and Biomedical Sciences, University of Bern, CH-3000 Bern, Switzerland
[5]Experimental Imaging Center, San Raffaele Scientific Institute, I-20132 Milan, Italy
[6]School of Life Sciences, École Polytechnique Fédérale de Lausanne, CH-1015 Lausanne, Switzerland
*co-first

maurizio.molinari@irb.usi.ch

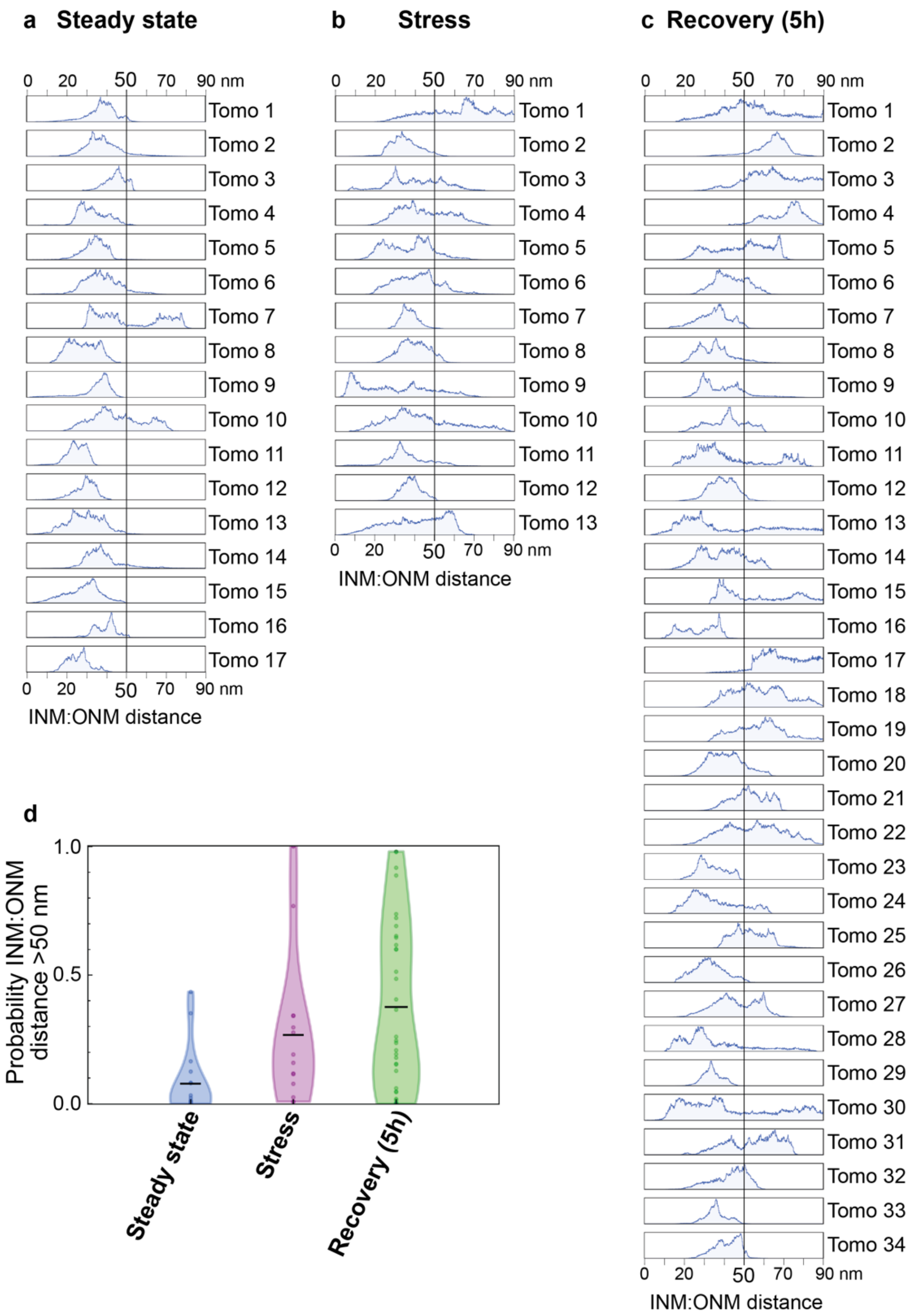

Supplementary Figure 1 – related to Figure 1

a The distance between INM and ONM was measured along segmented INM and ONM in 17 tomograms of nuclear envelope (collected from MEF at steady state).

b Same as **a** for 13 tomograms (pharmacologic induced ER stress).

c Same as **a** for 34 tomograms (images of nuclei collected 5 h after interruption of the CPA treatment).

d Violin plots showing the probability to observe a INM:ONM larger than 50 nm at steady state, during CPA-induced ER stress and 5 h after interruption of the CPA treatment.

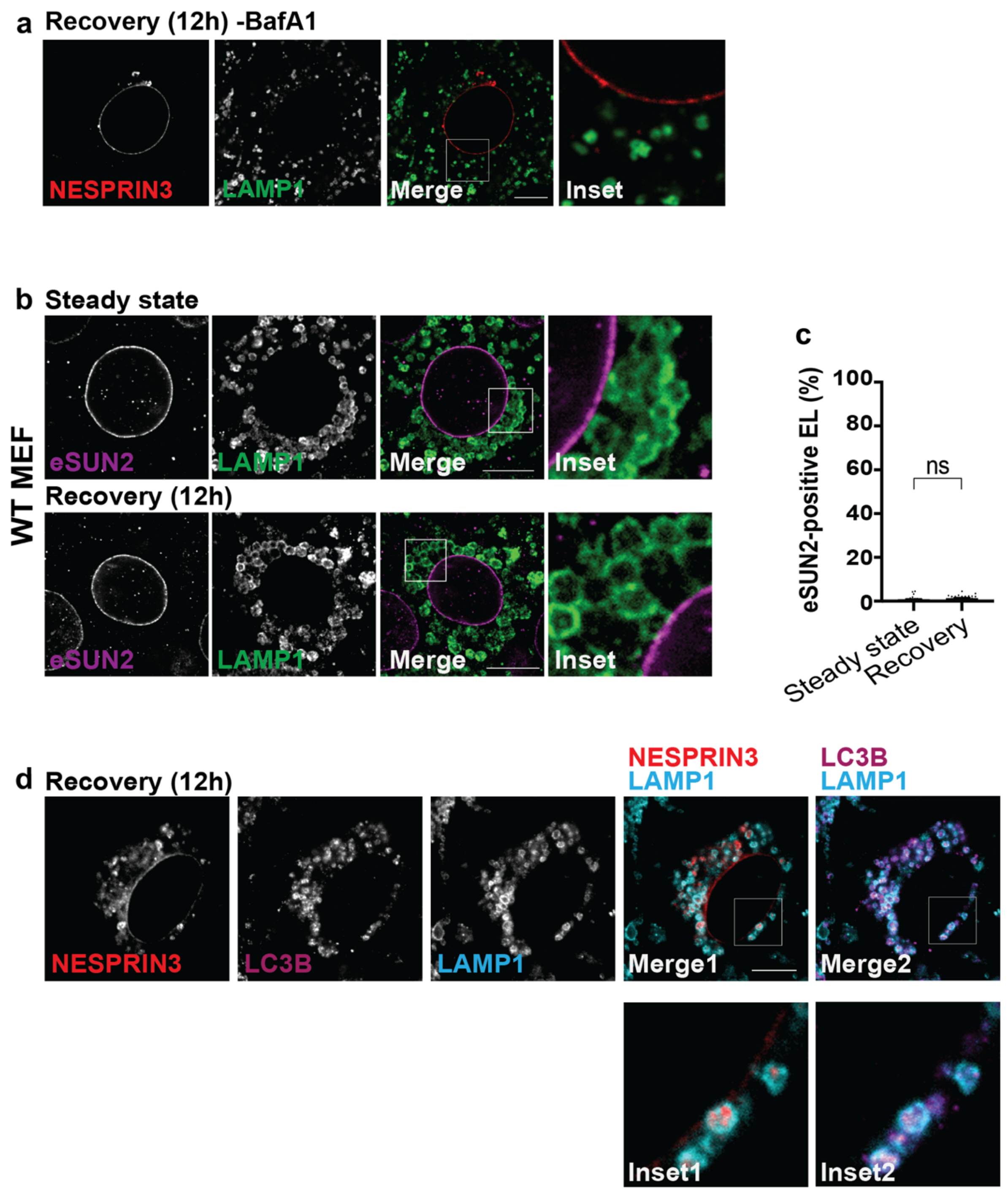

Supplementary Figure 2 – related to Figures 3

a Same as **Fig. 3a**, lower panel (Recovery), without BafA1. Scale bars: 10 µm. Experiment was performed once.

b CLSM analyses of subcellular localization of endogenous SUN2-positive INM subdomains delivery within LAMP1-positive endolysosomes in MEF at steady state (upper panels) or in MEF recovering from ER stress, 12 h after interruption of the pharmacologic treatment with CPA (lower panels). Scale bars: 10 µm.

c Quantification of **b** by LysoQuant. n=21 and 25 cells for steady state and recovery, respectively. N=2 independent experiments. mean ± SEM; unpaired, two-tailed t test, ns. P>0.5.

d Same as **b**, during recovery, to show the co-localization of LC3B and HALO-NESPRIN3α within LAMP1-positive endolysosome. Scale bars: 10 µm.

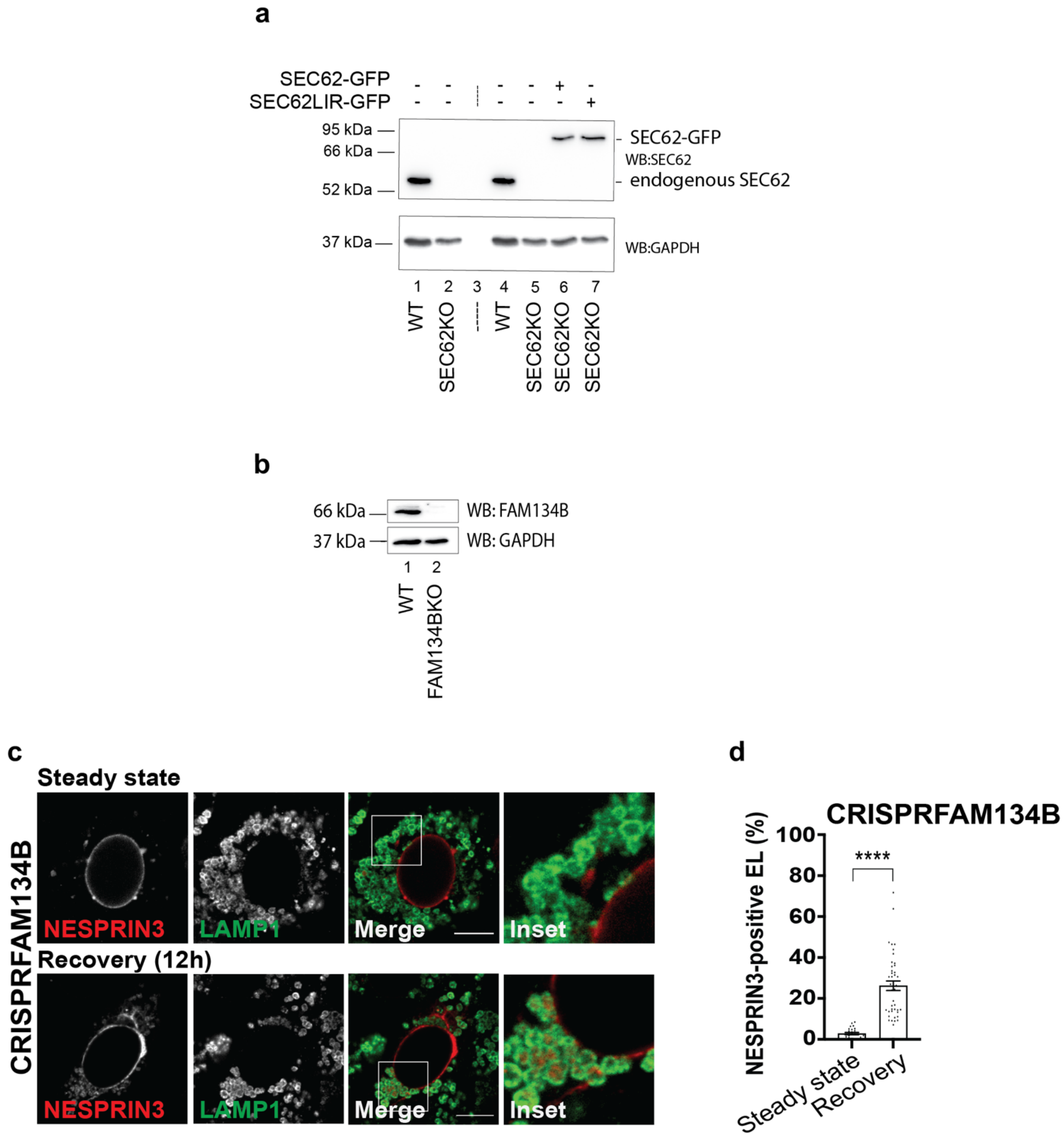

Supplementary Figure 3 – related to Figure 6

a WB analysis showing endogenous level of SEC62 in WT MEF (lanes 1 and 4), in MEF upon CRISPR/Cas9 genome editing to knock out SEC62 (lanes 2, 5-7), in SEC62-KO cells after back-transfection with SEC62-GFP (lane 6) or SEC62LIR-GFP, which cannot engage LC3 (lane 7). Scale bars: 10 µm. Uncropped blots in **SFig. 6**.

b Control of FAM134B knockout as obtained in MEF upon CRISPR/Cas9 genome editing.

c Unperturbed delivery of HALO-NESPRIN3α within LAMP1-positive endolysosomes in cells lacking the starvation-activated ER-phagy receptor FAM134B. Scale bars: 10 µm.

d Quantification of **c**. n=24 and 42 cells for steady state and recovery, respectively. N=2 independent experiments, mean ± SEM; unpaired, two-tailed t test, ****P< 0.0001.

WB data for CRISPRSEC62 and CRISPRFAM134B confirm data shown in Fumagalli et al, 2016 Fregno et al, 2018 and Loi et al 2019.

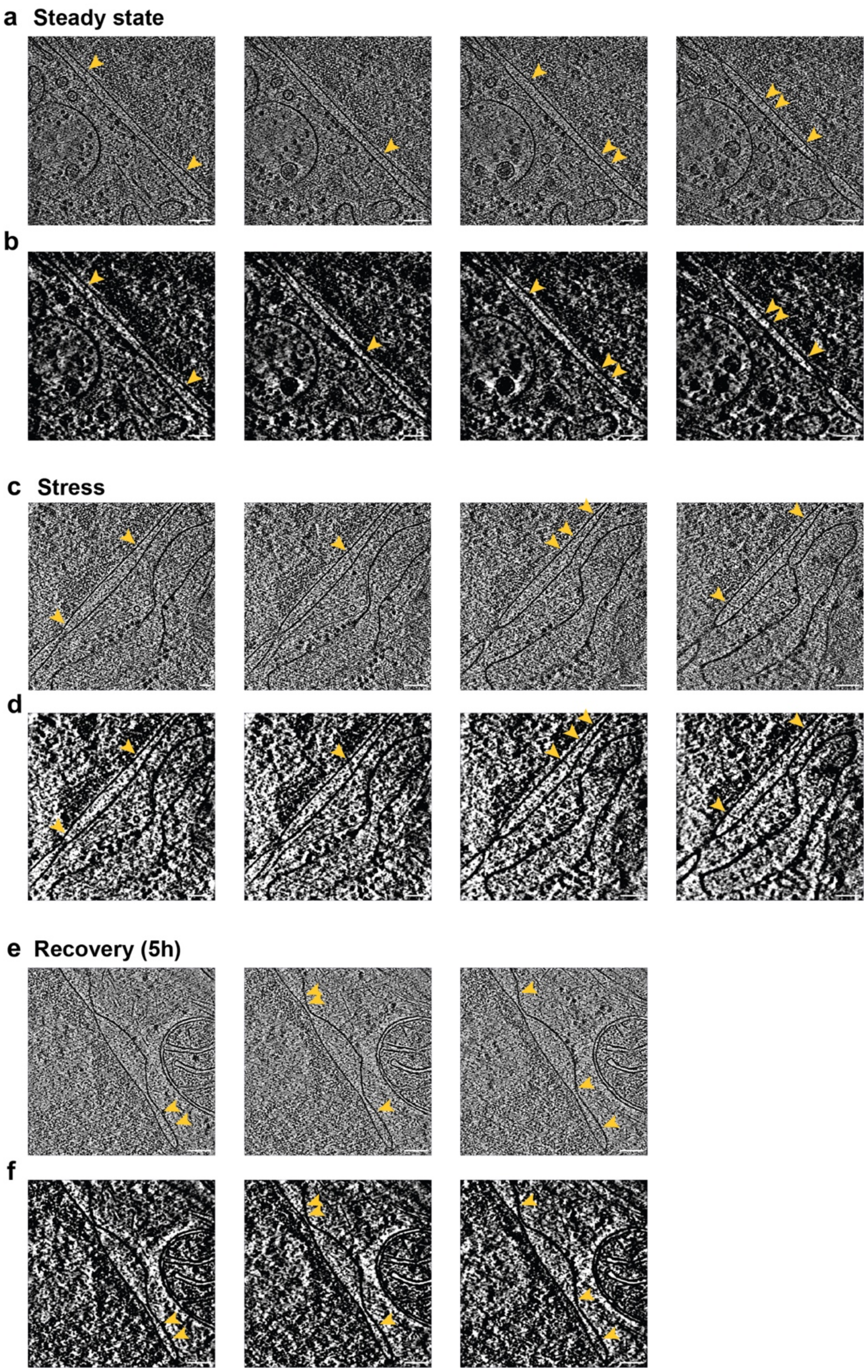

Supplementary Figure 4 – related to Figure 6
a Slices through tomograms on steady state cells.
b Corresponding slices in deconvoluted tomograms. Golden arrowheads indicate the position of continuous density filaments between ONM and INM as detected with density threshold mask in Avizo.
c Same as in **a** under ER stress.
d Same as in **b** under ER stress.
e Same as in **a** in MEF recovering from ER stress.
f Same as in **b** in MEF recovering from ER stress.
Scale bar 100 nm.

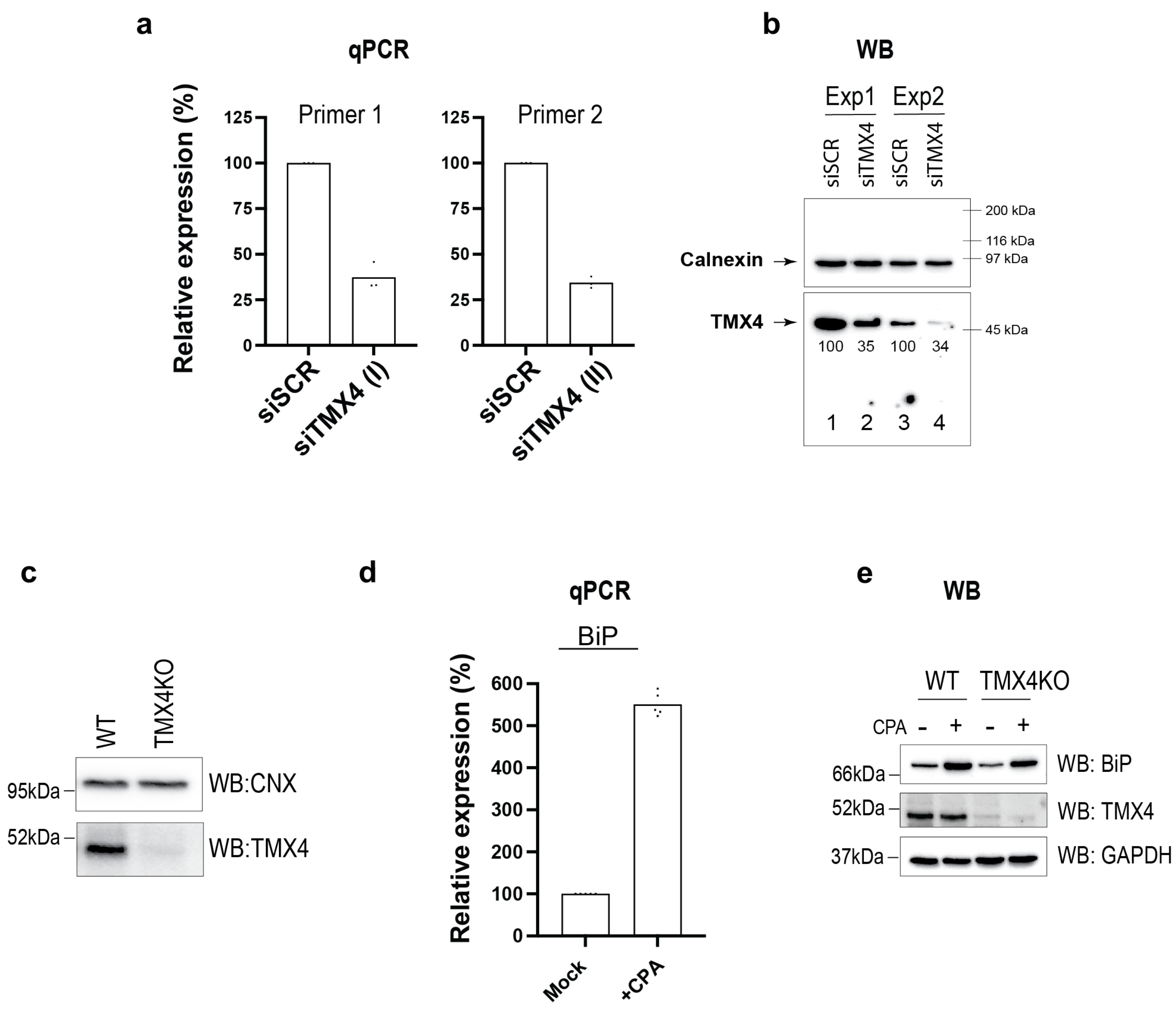

Supplementary Figure 5 – related to Figure 10

a Control of TMX4 silencing obtained by specific siRNAs showing a 70% reduction of TMX4 transcript as determined by qPCR with two different TMX4-specific primers.

b Same as **a** to control reduction of TMX4 at the protein level in two separate experiments (lower panel). In the western blots, the ER-resident protein calnexin is shown as loading control (upper panel). Relative intensity of the TMX4 protein bands is given. Uncropped blots in **SFig. 6**.

c Western blots to verify the TMX4 knockout obtained by CRISPR/Cas9 genome editing (calnexin is shown as loading control).

d TMX4 knockout cells respond to CPA-induced ER stress by substantially increase transcription of the conventional UPR marker BiP/GRP78 as determined by qPCR.

e Same as **d**, where induction of BiP/GRP78 is shown at the protein level. Note that the level of induction of BiP/GRP78 in wild type MEF and in TMX4-KO MEF is similar. GAPDH is shown as loading control.

WB are representative of at least two independent experiments, up to four times.

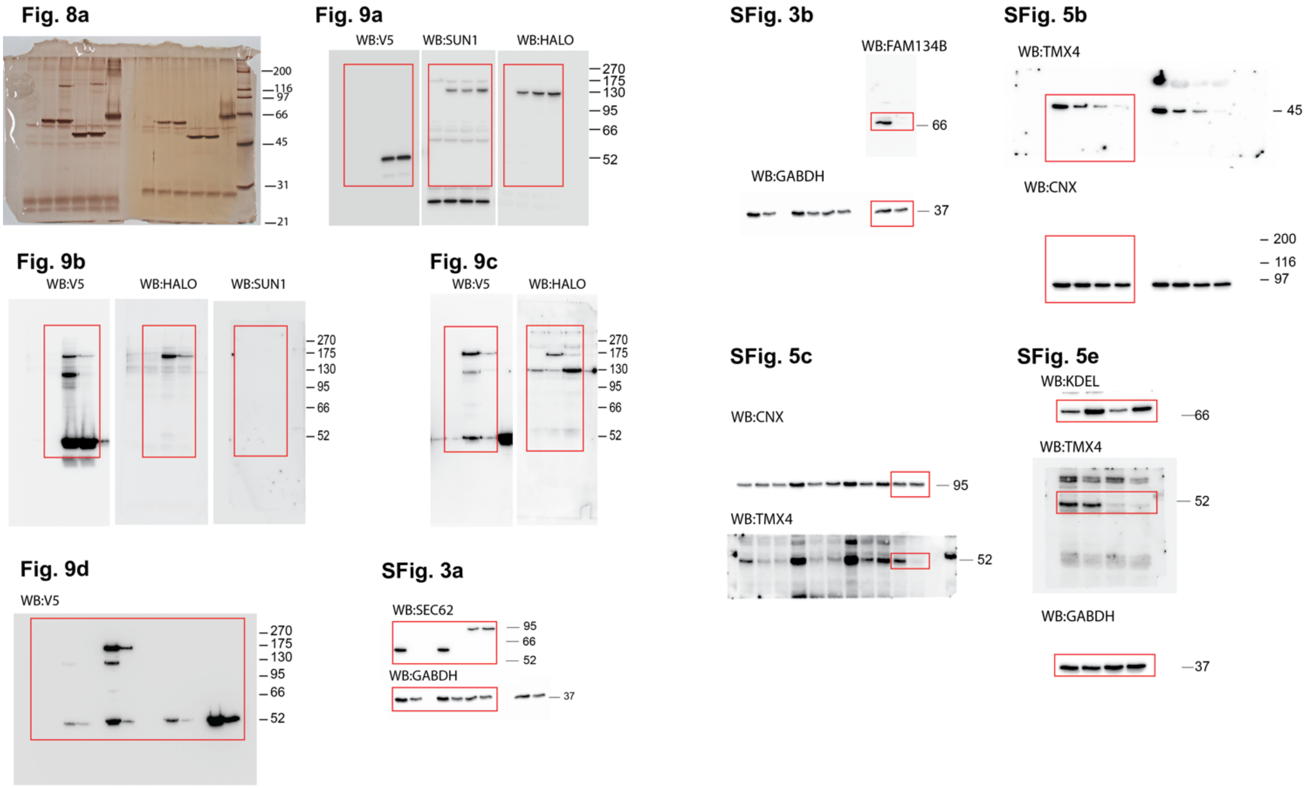

Supplementary Figure 6 – Uncropped gels and western blots

Identified Proteins	Accession Number	Alternate ID	Molecular Weight	pcDNA3.1	TMX3C/A	TMX4C/A	TMX5
Nesprin-2 OS	SYNE2_HUM	SYNE2	796 kDa	0	0	53	0
Neuropathy t	PLPL6_HUMA	PNPLA6	151 kDa	0	3	33	0
Thioredoxin-r	TMX4_HUMA	TMX4	39 kDa	0	0	21	4
Ectonucleoti	ENPP1_HUM	ENPP1	105 kDa	0	0	21	0
4F2 cell-surfa	4F2_HUMAN	SLC3A2	68 kDa	0	0	17	0
Protein disul	PDIA3_HUMA	PDIA3	57 kDa	0	10	15	13
Nesprin-1 OS	SYNE1_HUM	SYNE1	1011 kDa	0	0	14	0
Polymeric im	PIGR_HUMA	PIGR	83 kDa	0	0	11	0
Heterogeneo	HNRPU_HUN	HNRNPU	91 kDa	0	12	8	3
Tubulin beta	TBB4B_HUM	TUBB4B	50 kDa	0	8	8	11
Glutathione	GGT7_HUMA	GGT7	70 kDa	0	0	7	0
ATP synthase	ATPA_HUMA	ATP5F1A	60 kDa	0	5	5	0
Metal transp	CNNM3_HUI	CNNM3	76 kDa	0	3	5	0
Protein disul	PDIA1_HUMA	P4HB	57 kDa	0	2	5	17
Protein disul	TMX3_HUMA	TMX3	52 kDa	0	206	4	2
Dolichyl-diph	STT3A_HUM	STT3A	81 kDa	0	10	4	0
Heterogeneo	HNRPL_HUM	HNRNPL	64 kDa	0	8	4	3
Heat shock 7	HS71A_HUM	HSPA1A	70 kDa	0	5	4	9
Thioredoxin (	TXD15_HUM	TXNDC15	40 kDa	0	0	4	134
HLA class I hi	1C07_HUMA	HLA-C	41 kDa	0	0	4	0
Immunoglob	IGSF3_HUMA	IGSF3	135 kDa	0	0	4	0
Hemoglobin	HBB_HUMAN	HBB	16 kDa	0	0	4	0
X-ray repair (	XRCC5_HUM	XRCC5	83 kDa	0	5	3	0
WD40 repea	SMU1_HUMA	SMU1	58 kDa	0	2	3	0
Lactotransfe	TRFL_HUMA	LTF	78 kDa	0	0	3	0
Polypeptide I	GLT13_HUM	GALNT13	64 kDa	0	0	3	0
Polypeptide I	GALT1_HUM	GALNT1	64 kDa	0	0	3	0
Immunoglob	IGLC2_HUMA	IGLC2	11 kDa	0	0	3	0
Immunoglob	KV320_HUM	IGKV3-20	13 kDa	0	0	3	0
NPC intracell	NPC1_HUMA	NPC1	142 kDa	0	0	3	0
Mucin-5B OS	MUC5B_HUN	MUC5B	596 kDa	0	0	3	0
Protein FAM	F234B_HUM	FAM234B	67 kDa	0	0	3	0
Aspartate--tI	SYDC_HUMA	DARS	57 kDa	0	3	2	0
DNA replicat	MCM7_HUM	MCM7	81 kDa	0	2	2	0
Secretoglobi	SG1D2_HUM	SCGB1D2	10 kDa	0	2	2	0
Poly [ADP-rib	PARP1_HUM	PARP1	113 kDa	0	2	2	0
Transcriptior	GATA4_HUM	GATA4	45 kDa	0	2	2	0
WW domain	WBP1L_HUN	WBP1L	38 kDa	0	0	2	0
Probable ATF	DDX5_HUMA	DDX5	69 kDa	0	0	2	0
V-type proto	VAS1_HUMA	ATP6AP1	52 kDa	0	0	2	0
Scavenger re	SCRB1_HUM	SCARB1	61 kDa	0	0	2	0
RNA-binding	EWS_HUMA	EWSR1	68 kDa	0	0	2	0
ATP-depende	RECQ1_HUM	RECQL	73 kDa	0	0	2	0
Protein LTV1	LTV1_HUMA	LTV1	55 kDa	0	0	2	0
Phospholipas	PLD3_HUMA	PLD3	55 kDa	0	0	2	0
Integrin beta	ITB1_HUMAI	ITGB1	88 kDa	0	0	2	0
Protein MAL	MAL2_HUMA	MAL2	19 kDa	0	0	2	0
Histone-argi	CARM1_HUN	CARM1	66 kDa	0	0	2	0
ER membrar	EMC1_HUMA	EMC1	112 kDa	0	0	2	0
Protocadheri	PCDB2_HUM	PCDHB2	87 kDa	0	0	2	0

Supplementary Table 1

Output of the MS analysis showing cellular polypeptides engaged in mixed disulfides with TMX3, TMX4 or TMX5. The table shows, at the top, the proteins most abundantly trapped in mixed disulfides with TMX4$_{C67A}$. In red, NESPRIN2 (SYNE2) and NESPRIN1 (SYNE1). The number reported is the total number of tandem mass spectra positively matched to the protein group. The full mass spectrometry proteomics data have been deposited to the ProteomeXchange Consortium via the PRIDE partner repository with the dataset identifier PXD041156 and 10.6019/PXD041156.
