# Supplementary material for: TMX4-driven LINC complex disassembly and asymmetric autophagy of the nuclear envelope upon acute ER stress
Source: Nat Commun. 2023 Jun 13;14:3497. doi: 10.1038/s41467-023-39172-3 (PMC10264389; doi:10.1038/s41467-023-39172-3)
Supplement: Supplementary file 9 — Reporting Summary [file 41467_2023_39172_MOESM9_ESM.pdf]

nature portfolio

Corresponding author(s): Maurizio Molinari

Last updated by author(s): 2023/05/16

Reporting Summary

Nature Portfolio wishes to improve the reproducibility of the work that we publish. This form provides structure for consistency and transparency in reporting. For further information on Nature Portfolio policies, see our Editorial Policies and the Editorial Policy Checklist.

Statistics

For all statistical analyses, confirm that the following items are present in the figure legend, table legend, main text, or Methods section.

n/a	Confirmed	
☐	☒	The exact sample size (*n*) for each experimental group/condition, given as a discrete number and unit of measurement
☐	☒	A statement on whether measurements were taken from distinct samples or whether the same sample was measured repeatedly
☐	☒	The statistical test(s) used AND whether they are one- or two-sided<br>*Only common tests should be described solely by name; describe more complex techniques in the Methods section.*
☒	☐	A description of all covariates tested
☒	☐	A description of any assumptions or corrections, such as tests of normality and adjustment for multiple comparisons
☐	☒	A full description of the statistical parameters including central tendency (e.g. means) or other basic estimates (e.g. regression coefficient) AND variation (e.g. standard deviation) or associated estimates of uncertainty (e.g. confidence intervals)
☐	☒	For null hypothesis testing, the test statistic (e.g. F, t, r) with confidence intervals, effect sizes, degrees of freedom and P value noted<br>*Give P values as exact values whenever suitable.*
☒	☐	For Bayesian analysis, information on the choice of priors and Markov chain Monte Carlo settings
☒	☐	For hierarchical and complex designs, identification of the appropriate level for tests and full reporting of outcomes
☒	☐	Estimates of effect sizes (e.g. Cohen's d, Pearson's r), indicating how they were calculated

Our web collection on statistics for biologists contains articles on many of the points above.

Software and code

Policy information about availability of computer code

Data collection	Immunoflurescence data: Leica TCS SP5 confocal system and Leica Stellaris SP8 microscope with Leica LAS X 4.5.0.025531<br>Western blotting data: FusionFX7 VILBER (Witec) with Fusion FX7 Edge 18.12 software<br>Electron microscopy:<br>Immunogold electron microscopy: Transmission Electron Microscope Talos L120C (FEI, Thermo Fisher Scientific) with a Ceta CCD camera (FEI, Thermo Fisher Scientific)<br>RT-Electron Tomography :Transmission Electron Microscope Talos L120C (FEI, Thermo Fisher Scientific) with Tomography 4.0 software (FEI, Thermo Fisher Scientific).<br>LC-MS data: Q-Exactive Plus mass spectrometer (Thermo Fisher Scientific) interfaced to a nanocapillary HPLC (Dionex RSLC 3000) and Xcalibur software (Tune 2.9, Thermo Fisher Scientific)<br>qPCR: QuantStudio™ 3 Real-Time PCR System<br>Cryo electron tomography: Talos Arctica (Thermo Fisher Scientific) with serialEM 3.8 software
Data analysis	ImageJ/Fiji 2.9.0<br>LysoQuant plugin for Fiji (an unbiased and automated deep learning tool for fluorescent image quantification, which is freely available (https://www.irb.usi.ch/lysoquant/))<br>IMOD 4.11<br>Microscopy Image Browser 2.802<br>Graphing and statistic analysis were performed using GraphPad PRISM 9.1.5.<br>Photoshop 24.2.0

Motioncor2
Matlab 2014a
IMOD 4.10.29
QuantStudio™ Design & Analysis Software 1.5.5
Mascot 2.6.2
Scaffold 4.9.0, Proteome Software Inc.
Excel 16.71
Avizo 9.2.0
Surface morphometrics 0.2
python 2.7.12
matplotlib2.2.5
UCSF Chimera 1.16

For manuscripts utilizing custom algorithms or software that are central to the research but not yet described in published literature, software must be made available to editors and reviewers. We strongly encourage code deposition in a community repository (e.g. GitHub). See the Nature Portfolio guidelines for submitting code & software for further information.

Data

Policy information about availability of data

All manuscripts must include a data availability statement. This statement should provide the following information, where applicable:

- Accession codes, unique identifiers, or web links for publicly available datasets
- A description of any restrictions on data availability
- For clinical datasets or third party data, please ensure that the statement adheres to our policy

The proteomics data generated in this study have been deposited in the ProteomeXchange under accession code PXD041156.

Human research participants

Policy information about studies involving human research participants and Sex and Gender in Research.

Reporting on sex and gender	N/A
Population characteristics	N/A
Recruitment	N/A
Ethics oversight	N/A

Note that full information on the approval of the study protocol must also be provided in the manuscript.

Field-specific reporting

Please select the one below that is the best fit for your research. If you are not sure, read the appropriate sections before making your selection.

☒ Life sciences ☐ Behavioural & social sciences ☐ Ecological, evolutionary & environmental sciences

For a reference copy of the document with all sections, see nature.com/documents/nr-reporting-summary-flat.pdf

Life sciences study design

All studies must disclose on these points even when the disclosure is negative.

Sample size	The number of cells analyzed and the number of independent experiments is specified for each experiment in the figure legends.
Data exclusions	No data were excluded from the analyses.
Replication	The number of cells analyzed and the number of independent experiments is specified for each experiment in the figure legends.
Randomization	No randomization was performed as this is not common for western blot analysis. Imaging aquisition/analysis was performed by at least two different scientist.
Blinding	Unbiased imaging data collection/analyses were performed by at least two different scientists.

Blinding	All conclusions in the study were made based on the statistical analyses.<br>The fluorescent image quantification were performed using LysoQuant, an unbiased and automated deep learning tool.

Reporting for specific materials, systems and methods

We require information from authors about some types of materials, experimental systems and methods used in many studies. Here, indicate whether each material, system or method listed is relevant to your study. If you are not sure if a list item applies to your research, read the appropriate section before selecting a response.

Materials & experimental systems

n/a	Involved in the study
☐	☒ Antibodies
☐	☒ Eukaryotic cell lines
☒	☐ Palaeontology and archaeology
☒	☐ Animals and other organisms
☒	☐ Clinical data
☒	☐ Dual use research of concern

Methods

n/a	Involved in the study
☒	☐ ChIP-seq
☒	☐ Flow cytometry
☒	☐ MRI-based neuroimaging

Antibodies

Antibodies used

LAMP1 DSHB HYBRIDOMA BANK clone 1D4B-c
V5 INVITROGEN, catalog number : R960-25
GFP ABCAM, catalog number : AB290
ANTI HALOTAG MONOCLONAL PROMEGA CORPORATION, catalog number : G921A,
TMX4 PROTEINTECH, catalog number : 21348-1-AP
KDEL STRESSGEN, catalog number : ADI-SPA-827 clone 10C3,
TEX264 NOVUS BIOLOGICALS, catalog number : NBP1-89866
SUN2 ABCAM, catalog number : AB124916 clone EPR6557
LC3B SIGMA ALDRICH, catalog number : L7543
GAPDH MILLIPORE, catalog number : MAB374, clone 6C5,
FAM134B GIFT FROM M. MIYAZAKI
CNX GIFT FROM A. HELENIUS
ERp57 GIFT FROM T.WILEMAN
SEC62 GIFT FROM R. ZIMMERMANN
PDI STRESSGEN, catalog number : PA890
ERp72 STRESSGEN, catalog number : SPA720

Anti-V5 Agarose Affinity Gel antibody SIGMA ALDRICH, catalog number : A7345
HALO-TRAP AGAROSE BEADS CHROMOTEK (PROTEINTECH), catalog number : OTA
GFP-TRAP AGAROSE BEADS CHROMOTEK (PROTEINTECH), catalog number : GTA
HRP-GOAT-ANTI MOUSE SOUTHERN BIOTECH , catalog number : 1031-05
PROTEIN A-HRP CONJUGATED INVITROGEN, catalog number : 10-1023

ALEXA FLUOR 647 GOAT ANTI RAT THERMO FISHER SCIENTIFIC, catalog number : A-21247
ALEXA FLUOR 568 GOAT ANTI RABBIT INVITROGEN, catalog number :A-11036
ALEXA FLUOR 405 GOAT ANTI RABBIT INVITROGEN, catalog number : A-31556
ALEXA FLUOR 488 DONKEY ANTI RABBIT THERMO FISHER SCIENTIFIC, catalog number : : A-21206
ALEXA FLUOR 488 GOAT ANTI MOUSE JACKSON IMMUNORESEARCH, catalog number : 115-545-166
ALEXA FLUOR 647 GOAT ANTI RABBIT JACKSON IMMUNORESEARCH, catalog number : LABS 111-605-144

NANOGOLD FAB GOAT ANTI RABBIT (H+L) NANOPROBES catalog number : 2004, lot#41C642
NANOGOLD FAB GOAT ANTI MOUSE (H+L) NANOPROBES catalog number : 2002, lot#09S212

Validation

The antibody used in this study from the commercial sources were validated according to the manufacturer`s protocol.
Non commercial antibodies for SEC62 and CNX were validated in previous work (Fumagalli et al, 2016). Antibodies validation: SEC62 (SFig.3A), FAM134B (SFig.3B), TMX4 (FSig.5C and SFig.5E), HALO (Fig.9A), GFP (Fig. 9A) and V5 (Fig. 9A).

Eukaryotic cell lines

Policy information about cell lines and Sex and Gender in Research

Cell line source(s)

MEF WT and Atg5KO are kind gift from N. Mizushima, MEF WT and Atg14KO are kind gift from T. Saitoh. CRISPRMOCK, CRISPRSEC62 and CRISPRFAM134B were generated by CRISPR/Cas9 genome editing in our lab as described previously (Fumagalli et al, 2016, Fregno et al, 2018, Loi et al 2019). NIH3T3 and HEK293 were purchased from Invitrogen and ATCC, respectively.

Authentication	Atg5KO MEF was a kind gift from N. Mizushima. ATG5KO was verified by immunoblot analysis of Atg5 and LC3 in Kuma et al. 2004.<br>Atg14KO MEF was a kind gift from T. Saitoh. Kageyama, S. et al. 2011<br>The identity of the ATG14KO cell line was checked by functional analyses (p62 turnover and LC3 lipidation on autophagy induction) in Loi et al 2019.<br>CRISPRSEC62 was verified by Western Blot with SEC62 antibody as described previously (Fumagalli et al, 2016, Fregno et al, 2018, Loi et al 2019) and in the Supplementary Figure 3a<br>CRISPRFAM134B was verified by Western Blot with FAM134B antibody as described previously (Fregno et al, 2018) and in the Supplementary Figure 3b
Mycoplasma contamination	The cell lines were tested regularly to be negative for mycoplasma contamination.
Commonly misidentified lines<br>(See ICLAC register)	Commonly misidentified lines were NOT used in this study.
